# Expression and Distribution of the Adrenomedullin System in Newborn Human Thymus

**DOI:** 10.1371/journal.pone.0097592

**Published:** 2014-05-15

**Authors:** Sara De Martin, Giovanna Paliuri, Annasandra Belloni, Genny Orso, Erica Zanarella, Giovanni Stellin, Ornella Milanesi, Giuseppe Basso, Ezia Maria Ruga, Chiara Frasson, Daniela Gabbia, Giada Perdoncin, Pietro Palatini, Sergio Bova

**Affiliations:** 1 Department of Pharmaceutical and Pharmacological Sciences, University of Padova, Padova, Italy; 2 Department of Molecular Medicine, University of Padova, Padova, Italy; 3 Scientific Institute IRCCS Eugenio Medea, Conegliano, Treviso, Italy; 4 Pediatric and Congenital Cardiac Surgery Unit, Department of Cardiac, Thoracic and Vascular Surgery, University of Padova, Padova, Italy; 5 Department of Pediatrics, University of Padova, Padova, Italy; Northwestern University Feinberg School of Medicine, United States of America

## Abstract

Adrenomedullin (AM) is a multifunctional peptide endowed with various biological actions mediated by the interaction with the calcitonin receptor-like receptor (CLR), which couples to the receptor activity-modifying proteins 2 or 3 (RAMP2 or RAMP3) to form the functional plasma membrane receptors AM1 and AM2, respectively. In this study, we investigated for the first time the expression and localization of AM, CLR, RAMP2 and RAMP3 in human thymic tissue from newborns and in primary cultures of thymic epithelial cells (TECs) and thymocytes. Immunohistochemical analysis of thymic tissue showed that both AM and RAMP2 are abundantly expressed in the epithelial cells of medulla and cortex, blood vessels and mastocytes. In contrast, RAMP3 could not be detected. In cultured TECs, double immunofluorescence coupled to confocal microscopy revealed that AM is present in the cytoplasmic compartment, whereas RAMP2 could be detected in the cytoplasm and nucleus, but not in the cell membrane. At variance with RAMP2, CLR was not only present in the nucleus and cytoplasm of TECs, but could also be detected in the cell membrane. The nuclear and cytoplasmic localizations of RAMP2 and CLR and the absence of RAMP2 in the cell membrane were confirmed by western-blot analysis performed on cell fractions. AM, RAMP2 and CLR could also be detected in thymocytes by means of double immunofluorescence coupled to confocal microscopy, although these proteins were not present in the whole thymocyte population. In these cells, AM and RAMP2 were detected in the cytoplasm, whereas CLR could be observed in the cytoplasm and the plasma membrane. In conclusion, our results show that the AM system is widely expressed in human thymus from newborns and suggest that both AM1 receptor components CLR and RAMP2 are not associated with the plasma membrane of TECs and thymocytes but are located intracellularly, notably in the nucleus.

## Introduction

The thymus provides a variety of specialized microenvironments that support the production of self-tolerant T cells starting from immature precursors [1]. As reviewed recently [2], each maturation event of T cell takes place in a discrete region of the thymus and relies on the interaction of thymocytes with specialized thymic epithelial cells (TECs), located in both the cortex (cortical thymic epithelial cells, cTECs) and the medulla (medullary thymic epithelial cells, mTECs). T cell progenitors enter the thymus at the cortex/medulla border via post–capillary venules and migrate toward the capsule in response to chemokine signalling. In the cortex, thymocytes undergo positive selection by cTECs and then migrate to the medulla where they are screened for reactivity to tissue-restricted self antigens expressed by mTECs [3]. Developing thymocytes and TECs establish a mutual “cross talk” that is necessary for the functional maturation of both types of cells [2]. Mature T cells exit the thymus via blood or lymphatic vessels in response to a sphingosine-1-phosphate (S1P) gradient [4]. Thymic functions are regulated by various peptides, such as ghrelin [5], leptin [6],[7], neuronal growth factor [8], [9] and interleukins [10].

Adrenomedullin (AM) is a multifunctional peptide which exerts, through an autocrine/paracrine mode of action, multiple biological effects [8], including the regulation of blood pressure, cell growth and differentiation, modulation of hormone secretion, central nervous system functions and the potentiation of host defences against microbes [11], [12]. AM exerts its biological effects by interacting with a functional receptor formed by the combination of the calcitonin receptor-like receptor (CLR), a 7-transmembrane domain G protein-coupled receptor (GPCR), with a family of receptor activity-modifying proteins (RAMPs) that dictate its ligand binding specificity. The association of CLR with RAMP1 results in a receptor that binds preferentially to calcitonin gene-related peptides (CGRP), whereas the association of CLR with RAMP2 or RAMP3 confers preferential AM binding [13]. AM binding to its receptors leads to adenylate cyclase activation, resulting in intracellular cAMP elevation [14], [15].

In previous studies we provided the first demonstration that the AM system is expressed in rat thymus, where it may play a role in thymus growth and thymocyte differentiation [16], [17]. To our knowledge, no data are available regarding the presence of the AM system in the human thymus. Therefore, we investigated the expression and distribution of AM, CLR, RAMP2 and RAMP3 in the human thymus of newborns undergoing open heart surgery. Newborns were chosen because we previously observed that the AM system expression was markedly higher in the thymus of newborn than adult rats [16]. In this study, we also investigated for the first time the subcellular localization of the AM system in TECs and thymocytes.

## Materials and Methods

After obtaining written informed consent from parents, human thymus fragments (1–2 cm^3^ ca.) were obtained as surgical tissue discards from 15 newborn patients undergoing cardiac surgery at the Paediatric Cardiosurgery Division of the University of Padova. The protocol was approved by the Ethics Committee of the University Hospital of Padova, Padova, Italy.

### Immunohistochemical analysis

Conventional immunohistochemistry was performed on paraformaldehyde-fixed sections, followed by the detection of the antibody as previously described [18]. Briefly, frozen sections (10 µm-thick) were cut with a cryostat (Leica CM-1850) at −20°C. Antigens were retrieved by heating the sections previously immersed in Bond Epitope Retrieval solution 1 (Vision Biosystem) at 100°C for 30 min. Sections were washed in phosphate buffered saline (PBS) and incubated with the following primary antibodies: mouse monoclonal anti-Ck 8/18 antibody (Santa Cruz Biotechnology, Inc. Santa Cruz, CA, USA), diluted 1∶200; mouse monoclonal anti-mast cell Tryptase antibody (Abcam, Cambridge, UK), diluted 1∶100; rabbit polyclonal anti-AM, anti-RAMP2 and anti-RAMP3 antibodies (all from Santa Cruz Biotechnology, Inc. Santa Cruz, CA, USA, diluted 1∶200). All antibodies were diluted in PBS containing 2% human serum and 2% normal goat serum. The chromogen reaction was developed using 3,3′-diaminobenzidine (DAB) substrate for Peroxidase (Sigma-Aldrich, Milan, Italy); other sections were immunolabeled with green and/or red fluorochrome-labeled antibody and finally counterstained with the nuclear dye 4′,6-diamidino-2-phenylindole (DAPI, Santa Cruz Biotechnology, Inc. Santa Cruz, CA, USA). For control purposes, the primary antibody was replaced by a non-immune normal goat serum, as well as by the use of primary antibodies pre-absorbed with antigen excess. Each experiment was conducted on thymi obtained from 5 different donors.

The observations were documented using light microscope Leica DMR equipped with Leica DC200/400 camera. Immunohistochemical examinations of thymic tissue were all performed by the same observer (ASB).

### Primary cultures of thymic epithelial cells

TEC cultures were obtained from human thymic fragments following a previously described method [19], with minor modifications. Briefly, thymic tissue was minced in small fragments, which were anchored in cell culture flasks and cultured in TEC medium [Minimum Dulbecco's Essential medium (Sigma-Adrich, Milan, Italy) enriched with 10% heat inactivated Fetal Bovine Serum (FBS) (Gibco, Life Technologies Italia, Monza, Italy), 100 ng/ml epidermal growth factor, 0.5 µg/ml hydrocortisone, 10 ng/ml cholera toxin (all from Sigma-Adrich, Milan, Italy), 2 mM glutamine, 100 units/ml penicillin, and 100 µg/ml streptomycin (all from Gibco, Life Technologies Italia, Monza, Italy). Cultures were maintained in a humidified atmosphere at 37°C and 5% CO_2_. Fragments were removed at day 14 or 21, and adherent cells were passed by tryptic digestion. Ck 8/18 staining was routinely performed to assess the purity of TEC cultures.

### Thymocyte isolation

Thymocytes were collected from Minimum Dulbecco's Essential medium immediately after thymic tissue fragmentation. Contaminating red blood cells were eliminated by treatment with Red blood cell lysing buffer (from Sigma-Adrich, Milan, Italy), according to manufacturer's instruction. Thymocytes were frozen and stored in liquid nitrogen until used.

### Immunocytochemical analysis

To evaluate the expression of cytokeratin 8/18, a selective marker of epithelial cells, TECs (at passage 2–4) were cultured on 12-mm diameter coverslips until reaching semi-confluence, when they were fixed in 4% paraformaldehyde (Electron Microscopy Sciences, Hatfield, PA, USA). Permeabilization of cell membranes and blocking of unspecific binding sites were performed by incubation with PBS containing 0.1% Triton and 0.2% bovine serum albumin (both from Sigma-Adrich, Milan, Italy). TECs were then incubated overnight at 4°C with a mouse monoclonal antibody anti-Ck 8/18 (Santa Cruz Biotechnology, Inc. Santa Cruz, CA, USA), diluted 1∶200 in PBS containing 2% human serum and 2% normal goat serum. After being washed, cells were incubated for 180 minutes at room temperature with an anti-mouse horseradish peroxidase (HRP)-conjugated secondary antibody (Sigma-Aldrich, Milan, Italy). The chromogen reaction was developed using a DAB substrate for Peroxidase. For control purposes, the primary antibody was replaced by a non-immune normal goat serum. The observations were documented using light microscope Leica DMR equipped with Leica DC200/400 camera. Image acquisitions were all performed by the same observer (ASB). This experiment was performed on all thymi from which cell cultures were obtained (n = 10).

### Double immunofluorescence

RAMP2, CLR and AM expressions were evaluated in TECs and in TECs cocultured with thymocytes obtained from the same thymus. In the latter case, the two cell lines were mixed together 24 hours before the immunocytochemical analysis, in a thymocyte medium (Minimum Dulbecco's Essential medium enriched with 15% heat inactivated FBS, 2 mM glutamine, 100 units/ml penicillin, 100 µg/ml streptomycin). TECs (at passage 2–4) were cultured on 12-mm diameter coverslips until reaching semi-confluence, and were then fixed in 4% paraformaldehyde. Permeabilization of cell membranes and blocking of unspecific binding sites were performed by incubating the cells with phosphate buffered saline (PBS) containing 0.1% Triton and 10% FBS. TECs were then incubated 60 minutes at 37°C with a mouse monoclonal antibody anti-RAMP2 (Santa Cruz Biotechnology, Inc. Santa Cruz, CA, USA, dilution 1∶200) and a rabbit polyclonal antibody directed against either AM or CLR (both from Santa Cruz Biotechnology, Inc. Santa Cruz, CA, USA, used at dilution 1∶200). Control experiments were also performed in which a rabbit polyclonal antibody directed against a different epitope of RAMP2 (Abcam, Cambridge, UK, dilution 1 µg/ml), was used instead of the mouse monoclonal antibody. After being washed, cells were incubated for 60 minutes at 37°C with an anti-mouse secondary antibody conjugated with DyLight 488 and an anti-rabbit secondary antibody conjugated with DyLight 405 (Jackson ImmunoResearch Laboratories, Inc., West Grove, PA, USA). For nuclear staining, cells were further incubated with propidium iodide, after treatment with a 2 mg/ml RNAse solution (both reagents were from Sigma-Aldrich, Milan, Italy). In order to confirm the nuclear localization and the lack of membrane localization of RAMP2, cells were incubated with a rabbit polyclonal antibody anti-acetyl-histone H3 (EMD Millipore Corporation, MA, USA) or the PKH67 fluorescent cell linker kit for general cell membrane labelling (Sigma-Aldrich, Milan, Italy). For membrane labelling, cells were treated for 1 min with the PKH67 solution, before their fixation with 4% paraformaldehyde, according to manifacturer's instructions. For control purposes, primary antibodies were replaced by PBS. Unless otherwise specified, confocal images were acquired through ×60 CFI Plan Apochromat Nikon objectives with a Nikon C1 confocal microscope and analysed using the NIS Elements software (Nikon). Image acquisitions were all performed by the same observer (EZ). Each experiment was performed on thymi obtained from 10 different donors.

### Determination of AM receptor expression by Western Blot

The analysis of CLR and RAMP2 protein expression was performed in nuclear and cytoplasmic fractions of TECs (passage 2–4), obtained by means of NE-PER (Nuclear and Cytoplasmic Extraction Reagents, Thermo Scientific, Rockford, IL, USA), following the manufacturer's instructions. The fractions, obtained from the cells of 10 different thymic fragments, were assessed for their protein concentration by means of the Novagen BCA Protein Assay kit (Novagen, Merck KGaA, Darmstadt, Germany), diluted to a protein concentration of 2 µg/µl and kept at 95°C for 5 minutes. Twenty µg of proteins per lane were subjected to sodium dodecylsulfate polyacrylamide gel electrophoresis (SDS-PAGE) on 10% polyacrylamide gels according to Laemmli [20] and then transferred to a 0.45 µm nitrocellulose membrane (Biorad Laboratories S.r.l., Segrate, Milan, Italy) at 250 mA for 90 minutes in the presence of 25 mM TRIS and 192 mM Glycine (both from Sigma-Aldrich, Milan, Italy). The membrane was blocked for unspecific binding sites with 10% dry milk in Tris-buffered saline containing 0.1% Tween 20, 150 mM NaCl (both from Sigma-Aldrich, Milan, Italy) and 10 mM TRIS (TBS-T). After being washed, the membrane was incubated overnight at 4°C with either a mouse monoclonal anti-RAMP2 antibody or a rabbit anti-CLR antibody, both diluted 1∶200 in 5% milk containing TBS-T. After washing, the membrane was incubated with a secondary anti-mouse or anti-rabbit antibody, conjugated with horseradish peroxidase. β-actin (Santa Cruz Biotechnology, Inc. Santa Cruz, CA, USA, dilution 1∶1000) was used as loading control. GAPDH and Lamin A/C (both from Santa Cruz Biotechnology, Inc. Santa Cruz, CA, USA, dilution 1∶500) were used to assess the purity of cytosolic and nuclear fraction, respectively. Reactive proteins were stained with the Amersham ECL Plus Western blotting detection system (GE Healthcare GmbH, Milan, Italy) and visualized with a VersaDoc MP 4000 instrument (Biorad Laboratories S.r.l.).

### cAMP assay

cAMP levels were determined by enzyme immunoassay (EIA) following the manufacturer's instructions (Cyclic AMP EIA Kit; Cayman Chemical Company, Ann Arbor, MI, USA) and according to the method of Schwarz et al. [21], with slight modifications. TECs (at passage 2–4) were cultured on 6-well plates until reaching confluence and then deprived of serum overnight, whereas fresh thymocytes were cultured in serum-free medium overnight. The next day cells were treated with various concentrations of AM (ranging from 10^−9^ to 10^−7^ M) for 10 minutes. Forskolin (FK, Sigma-Aldrich, Milan, Italy) 10^−4^ M was used as positive control. Absorbance was measured at 410 nm with a multilabel plate counter (Victor^2^-Wallac). Each experiment was performed in duplicate and repeated with cells obtained from 3 different donors. Data were analysed by means of the GraphPad Prism software, version 5 (GraphPad Software Inc., San Diego, CA, USA). Comparison of the experimental data obtained from control cell cultures and those treated with FK or different concentrations of AM was made by the non-parametric Kruskal-Wallis test. A P value <0.05 was considered statistically significant.

## Results

### AM and RAMP2 expression and distribution in human thymic tissue

**Figure 1:**
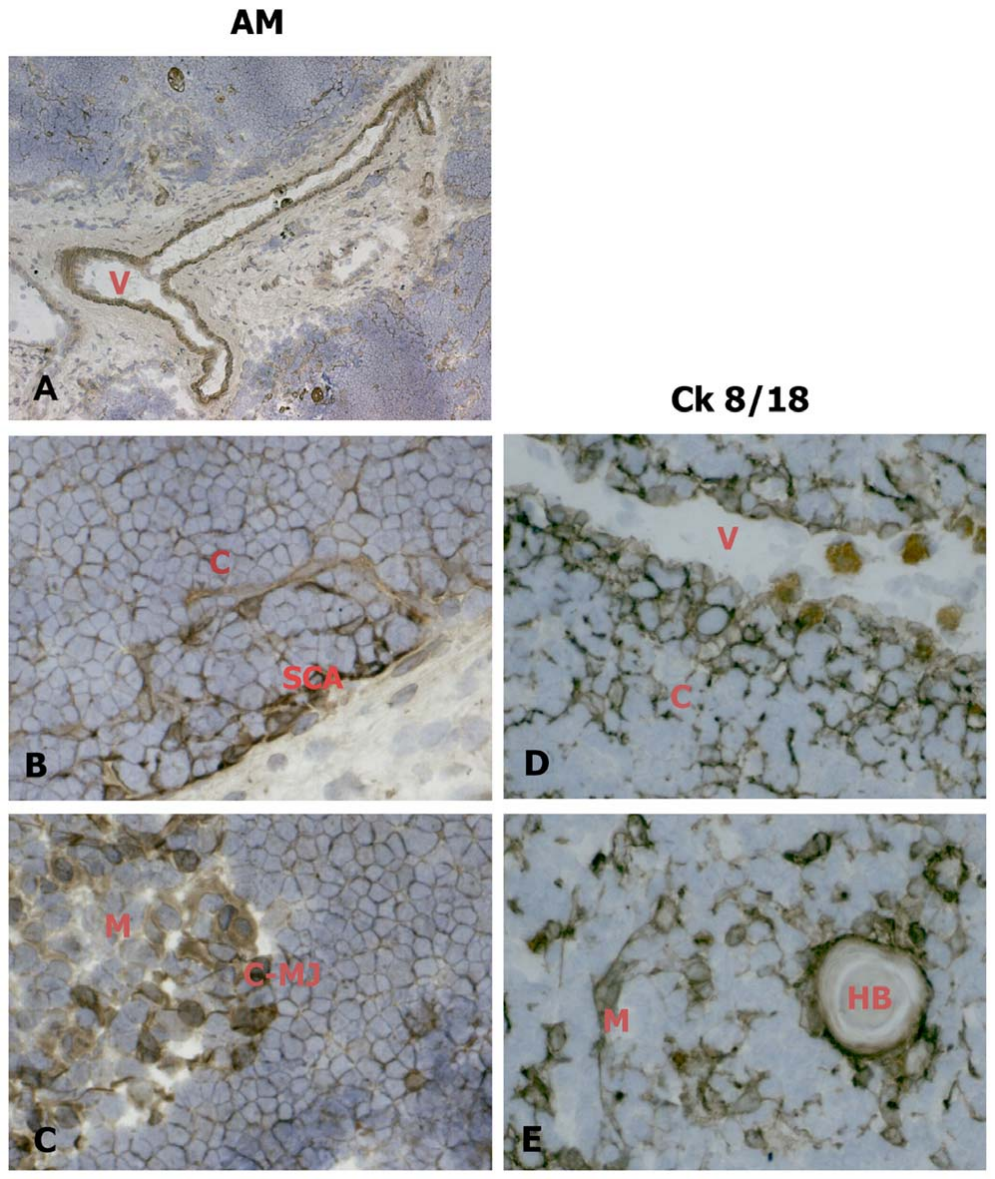
AM distribution in the epithelial compartment in cryostat sections of human newborn thymic tissue. Sections were incubated with an antibody against AM (left column) or Ck 8/18 (right column). AM expression in blood vessels (A). AM distribution is similar to that of Ck8/18 positive epithelial cells in the cortex (B, D), and the medulla (C, E). When, for control purposes, the primary antibody was replaced by a non-immune normal goat serum or by primary antibodies pre-absorbed with antigen excess, no reactivity was observed (not shown in the figure). Original magnification ×50 A; ×400 B, C, E, F. V: blood vessel; C: cortex; M: medulla; SCA: subcapsular area; C-MJ: cortico-medullary junction; HB: Hassal's body.

**Figure 2:**
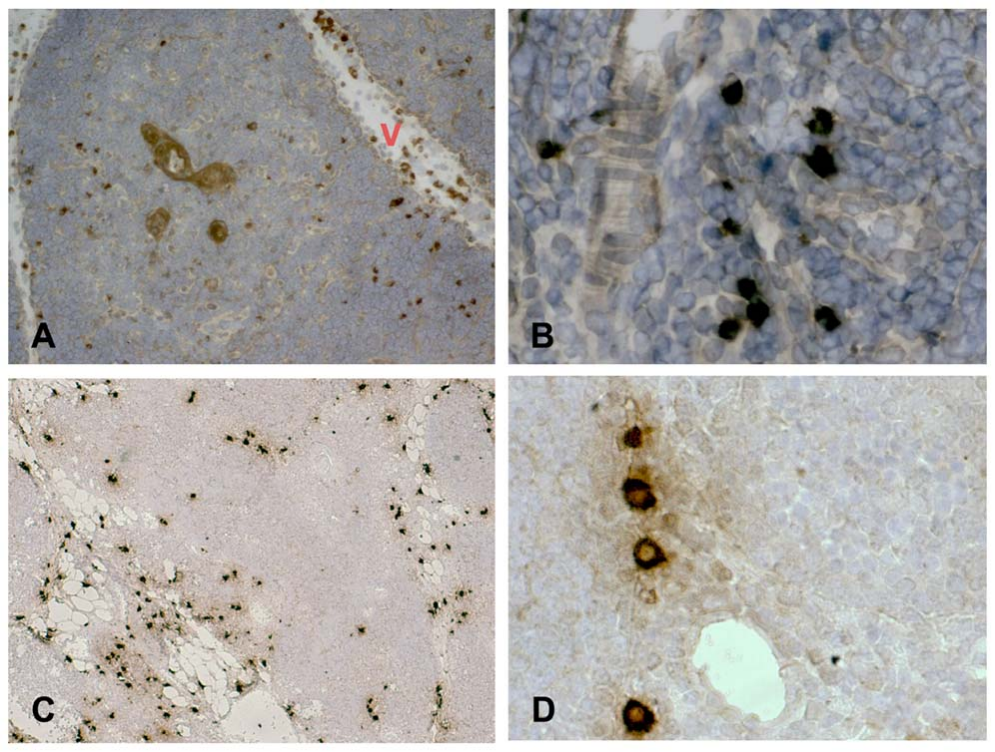
AM distribution in thymic mast cells in cryostat sections of human newborn thymic tissue. Sections were incubated with an antibody against tryptase (A, B) or AM (C, D). Mast cell were identified by tryptase immunopositivity. When, for control purposes, the primary antibody was replaced by a non-immune normal goat serum or by primary antibodies pre-absorbed with antigen excess, no reactivity was observed (not shown in the figure). Original magnification ×50 A, C; ×400 B, D. V: blood vessel.

Fig.1 shows that AM is diffusely distributed in the newborn thymus. AM immunoreaction was particularly evident in the media of thymic blood vessels (Fig. 1-A), the subcapsular TEC layer (Fig. 1-B) and the medulla (Fig. 1-C). No AM reaction was detected at the thymocyte level. A deeper analysis, employing an antibody directed against Cytokeratin 8/18 (Ck8/18), a marker of epithelial cells [22], revealed that AM distribution is similar to that of Ck 8/18 positive epithelial cells in the cortex, where it assumes a typical uniform tridimensional organization (Fig. 1-D), and in the medulla, where mTECs and the border of Hassal's corpuscles are always defined by labelled cells (Fig. 1-E). Figure 2 shows that AM distribution is similar to that of tryptase (a marker of thymic mast cells) in the parenchymal tissue. Mast cells are scattered in the connective tissue in association with blood vessels (Fig. 2-A) and, to a lesser extent, between parenchymal cells (Fig. 2-B). AM-immunopositive cells display analogous distribution in thymic tissue (Fig. 2-C and, at higher magnification, 2-D).

**Figure 3:**
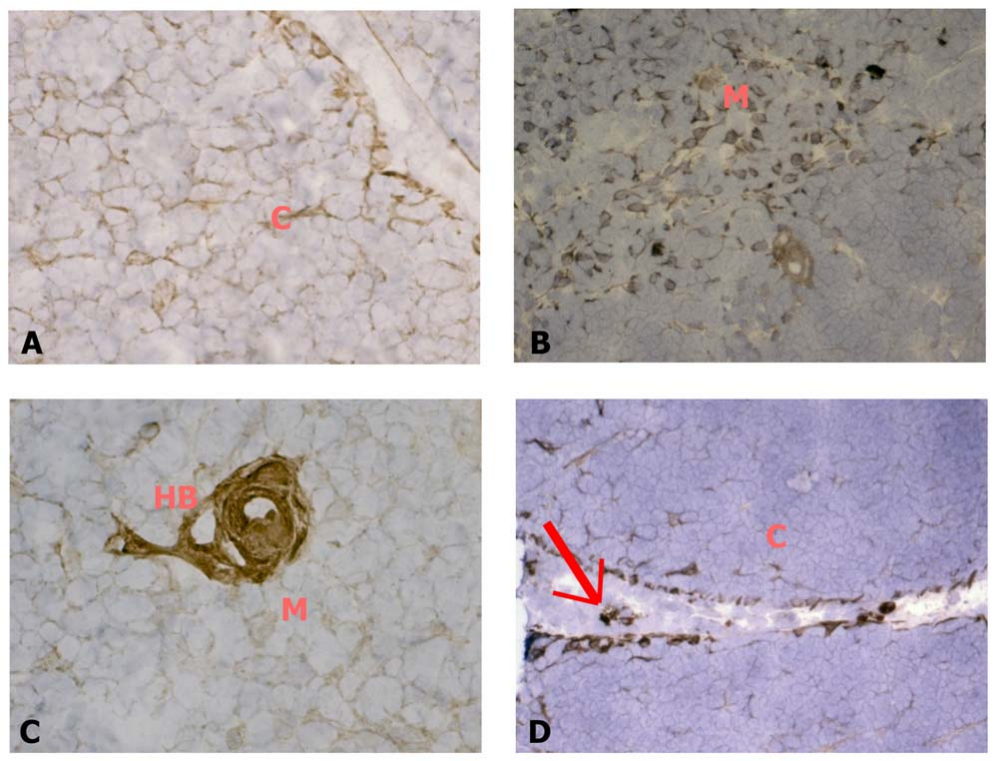
RAMP2 expression in cryostat sections of human thymic tissue. Sections were incubated with an antibody against RAMP2. RAMP2 is expressed in the cortical (A) and medullary (B) epithelial compartments, in Hassal's bodies (C) and in blood vessels and their related mastocytes (arrow in panel D). When, for control purposes, the primary antibody was replaced by a non-immune normal goat serum or by primary antibodies pre-absorbed with antigen excess, no reactivity was observed (not shown in the figure). Original magnification ×100. C: cortex; M: medulla; HB: Hassal's body.

RAMP2 immunopositivity is widely distributed in the human newborn thymus, especially in the epithelial compartments of the cortex (Fig. 3-A) and the medulla (Fig. 3-B), as well as in the Hassal's corpuscles (Fig. 3-C). RAMP2 is also expressed in blood vessels and their associated mastocytes (Fig. 3-D), which were detected by tryptase immunopositivity (data not shown). RAMP3 immunopositivity could never be observed in human thymic tissue (data not shown).

### Localization of AM and its receptor in cultured TECs and thymocytes

**Figure 4:**
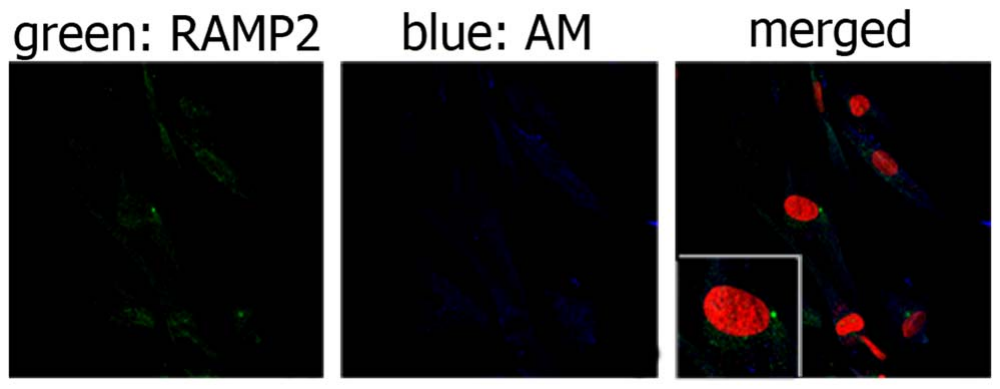
RAMP2 and AM distribution in cultured TECs. Immunofluorescence staining of TECs for RAMP2 (green) and AM (blue). RAMP2 displays a nuclear or perinuclear distribution and could not be seen in the cell membrane, whereas AM is widespread in the cytoplasmic compartment and seems to be located in vescicles. Cell nuclei are red stained with propidium iodide. When, for control purposes, the primary antibody was replaced by a non-immune PBS solution, no reactivity could be observed (not shown in the figure).

**Figure 5:**
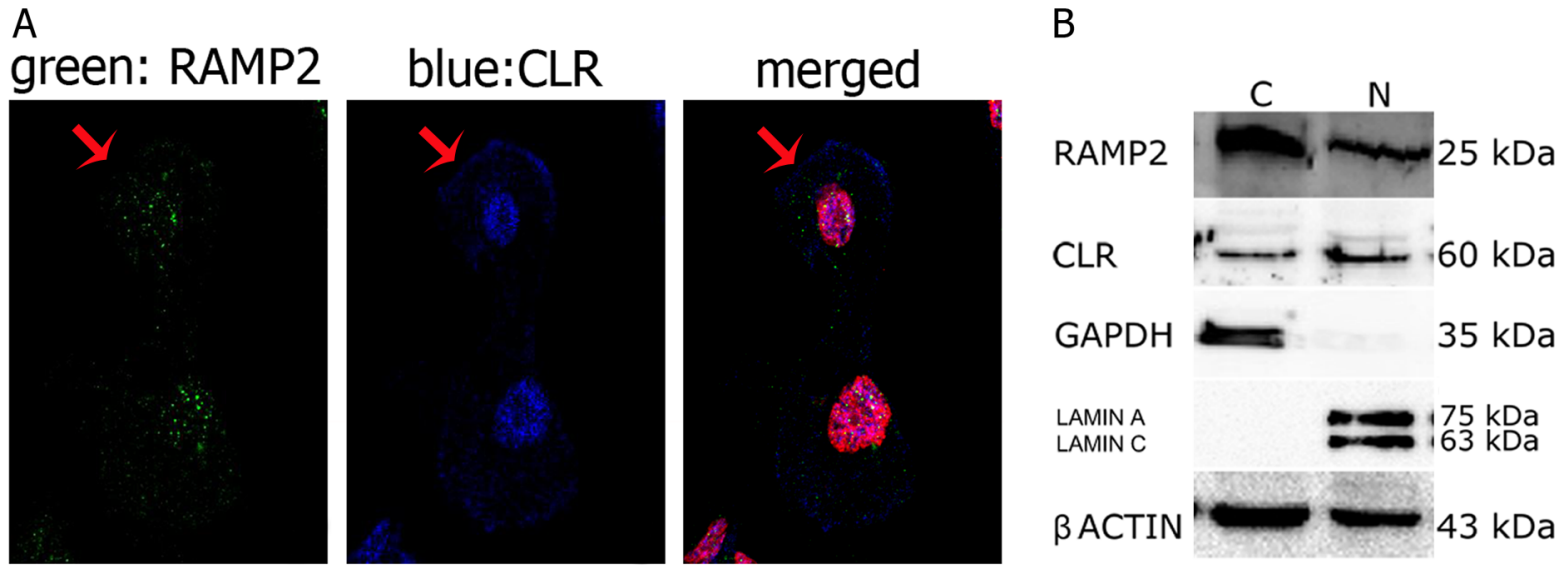
RAMP2 and CLR distribution in cultured TECs. (**A**) Immunofluorescence staining of TECs for RAMP2 (green) and CLR (blue). RAMP2 is evident inside the cell nucleus and cytoplasm, whereas CLR appears widespread in TECs, showing immunoreaction in nuclei, cytosol and cell membranes (arrow). Cell nuclei are red stained with propidium iodide. When, for control purposes, the primary antibody was replaced by a non-immune PBS solution, no reactivity could be observed (not shown in the figure). (**B**) A typical western blot analysis of RAMP2 and CLR. Protein expression was analyzed in the cytoplasmic (C) and nuclear (N) fractions of TECs. GAPDH and Lamin A/C were used as cytoplasmic and nuclear markers, respectively, to exclude contamination during cell fraction isolation. β-actin was used as loading control.

AM, CLR and RAMP2 cellular localization was investigated in cultured human TECs and thymocytes obtained from newborn thymi by double immunofluorescence coupled to confocal microscopy, and by western-blot analysis. Preliminary experiments were carried out to exclude the contamination of TEC cultures with fibroblasts by using an anti-Ck 8/18 antibody [22] (Fig. S1). Next, we evaluated the presence and localization of AM, RAMP2 and CLR in TECs. As illustrated in Fig. 4, both AM and RAMP2 were detected in TECs. In particular, AM was located in the cytoplasmic compartment, in which it was not uniformly distributed, but rather organized in clusters (blue fluorescence). RAMP2 reactivity (green fluorescence) was detected in the perinuclear and nuclear areas. We also evaluated the relative expression of RAMP2 and CLR, the two proteins forming the functional AM1 receptor. As shown in Fig. 5-A, both CLR and RAMP2 were distributed in the nucleus and the cytoplasm, whereas CLR could also be detected in the cell membrane of TECs. The nuclear localization of RAMP2 was confirmed by staining with an antibody obtained from a different provider and directed against a different RAMP2 epitope (see Methods), as clearly shown in Fig. S2.

The presence of RAMP2 and CLR in the cytoplasmic and nuclear areas, observed with confocal microscopy, was confirmed by a western blot analysis showing that both proteins were expressed in the cytoplasmic and nuclear fractions of TECs (Fig. 5-B). As confirmed by the lack of lamin A/C in the cytoplasm and of GAPDH in the nucleus [23], the procedure for cell fractionation gave pure cytoplasmic and nuclear fractions.

**Figure 6:**
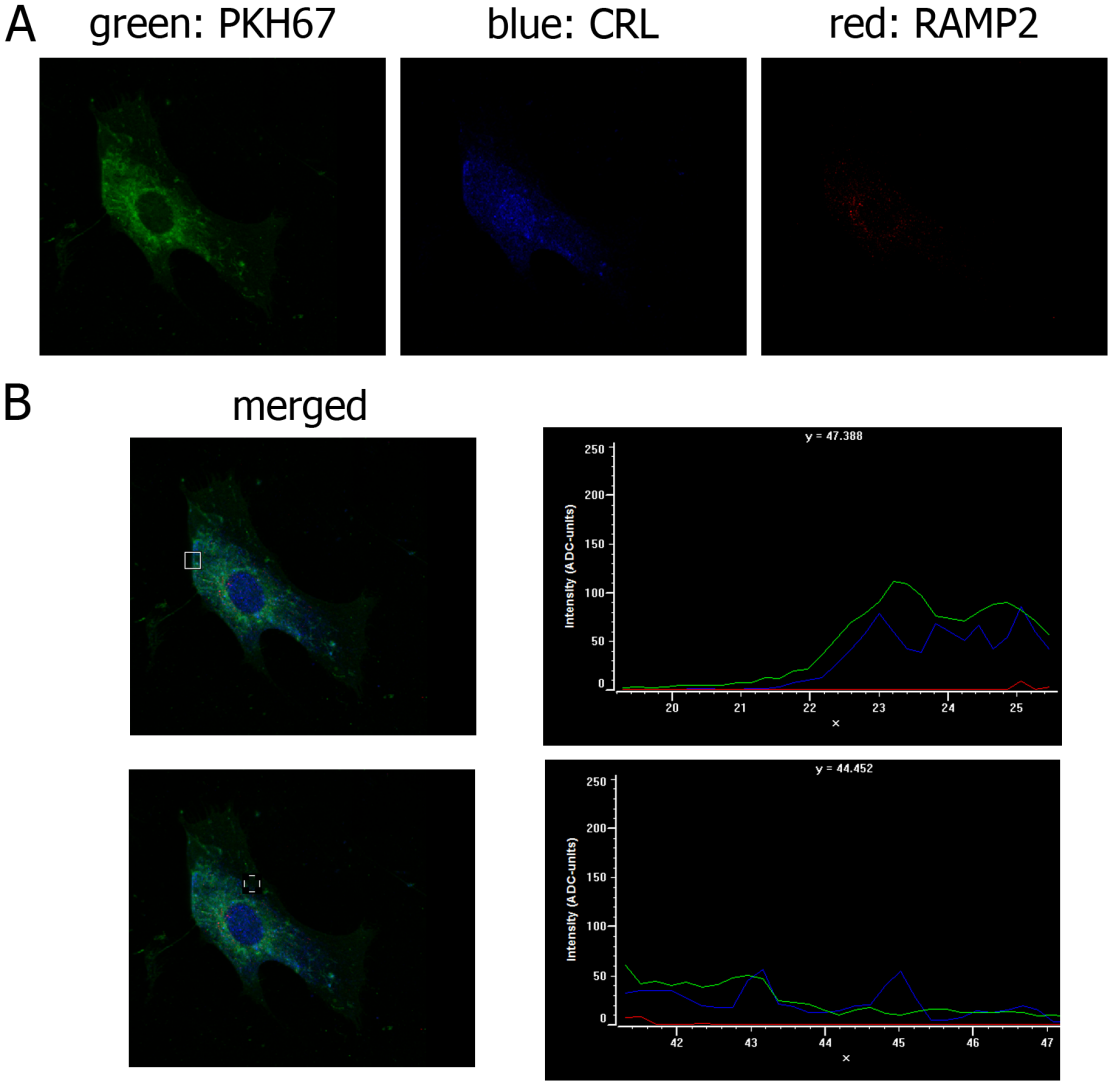
PKH67, CLR and RAMP2 distribution in cultured TECs. (**A**) Immunofluorescence staining of TECs for the plasma membrane marker PKH67 (green), CRL (blue) and RAMP2 (red). When, for control purposes, the primary antibody was replaced by a non-immune PBS solution, no reactivity could be observed (not shown in the figure). (**B**) Distribution of fluorescence intensity (ADC units) of PKH67 (green), CLR (blue) and RAMP2 (red) in two distinct plasma membrane regions (indicated by a square).

**Figure 7:**
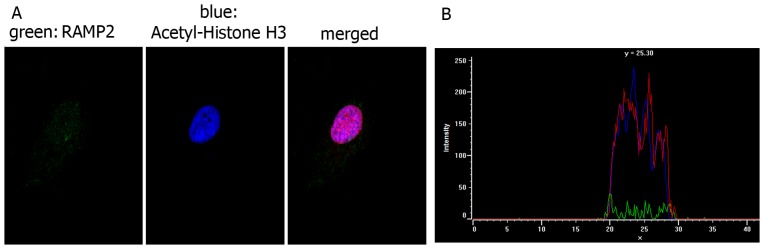
Acetyl-Histone H3 and RAMP2 distribution in cultured TECs. (**A**) Immunofluorescence staining of TECs for the nuclear marker Acetyl-Histone H3 (blue) and RAMP2 (green). Cell nuclei are red stained with propidium iodide. When, for control purposes, the primary antibody was replaced by a non-immune PBS solution, no reactivity could be observed (not shown in the figure). (**B**) Distribution of fluorescence intensity (ADC units) of RAMP2 (green), Acetyl-Histone H3 (blue) and propidium iodide (red). The three fluorescence signals display the same nuclear distribution.

The presence of CLR and the lack of RAMP2 in the plasma membrane were confirmed by the analysis of the co-localization of the two proteins with the plasma membrane marker PKH67 (Fig. 6-A). The intensity and localization of fluorescence was virtually identical for PKH67 and CLR, whereas RAMP2 could not be observed in the plasma membrane (Fig. 6-B). The nuclear localization of RAMP2 was confirmed by the analysis of co-localization with the nuclear markers acetyl-histoneH3 and propidium iodide. Fig. 7 shows that the fluorescent signals due to RAMP2 and the two nuclear markers have the same distribution.

**Figure 8:**
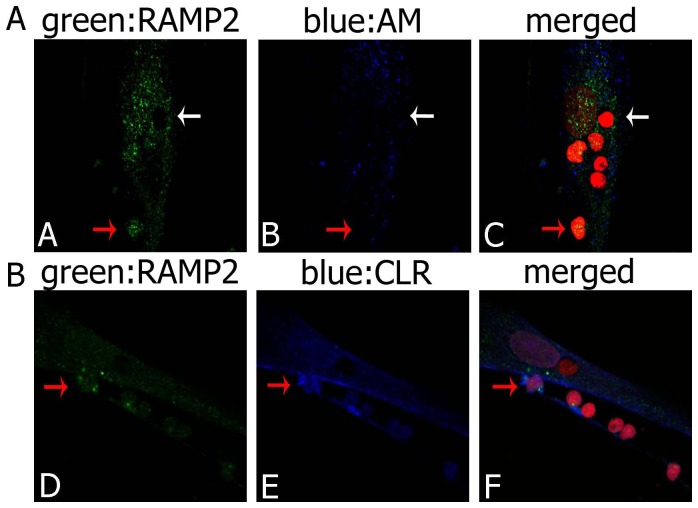
RAMP2, CLR and AM distribution in cultured thymocytes. (A) Immunofluorescence staining of thymocytes co-cultured with TECs for RAMP2 (green) and AM (blue). Only some thymocytes express RAMP2 and AM, mainly in the cytoplasmic compartment (one of them indicated by a red arrow), whereas other cells are not immunostained (white arrow). (B) Immunofluorescence staining of thymocytes co-cultured with TECs for RAMP2 (green) and CLR (blue). Co-presence of green and blue fluorescence is evident only in some thymocytes (red arrow). Cell nuclei are red stained with propidium iodide. When, for control purposes, the primary antibody was replaced by a non-immune PBS solution, no reactivity could be observed (not shown in the figure).

Confocal microscopy (Fig. 8) showed that AM, RAMP2 and CLR were expressed only in some thymocytes, in which AM immunopositivity was present in the cytoplasm (Fig. 8-A). RAMP2 (green) and CLR (blue) were usually observed in the cytoplasmic compartment, and, less frequently, in the nucleus; CLR was also detected in the plasma membrane (Fig. 8-B).

### Evaluation of cAMP levels in AM-stimulated TECs and thymocytes

**Figure 9:**
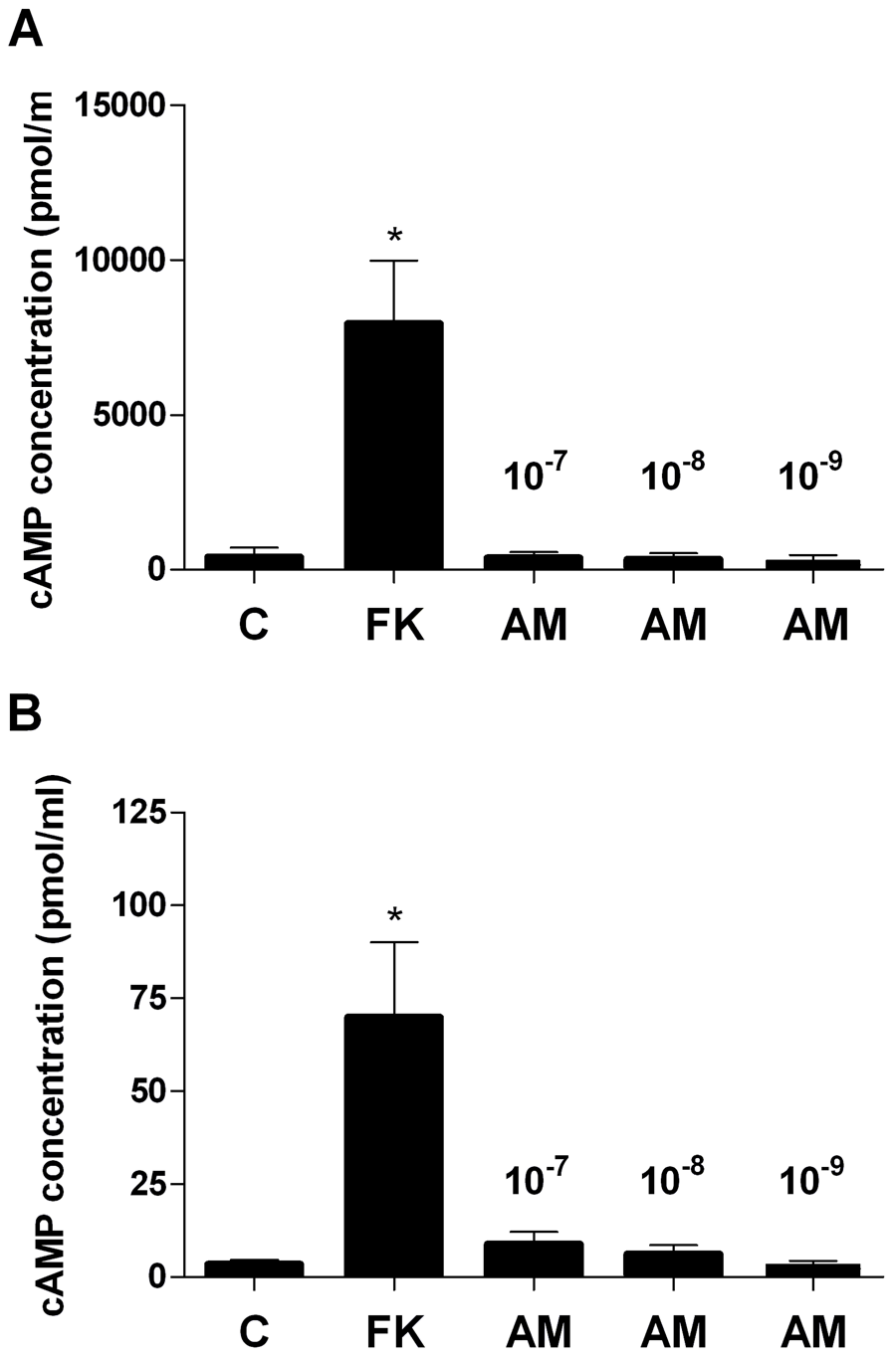
cAMP production in thymic cells. cAMP levels in TECs (A) and thymocytes (B) after exposure to AM or forskolin (FK). TECs and thymocytes were incubated with increasing concentrations of AM (ranging from 10^−9^ to 10^−7^). 10^−4^ FK was used as positive control. Values are means ± SD (n = 3). *P<0.05 *vs* control (C).

AM binding to its plasma membrane receptor AM1 induces the activation of adenylate cyclase with consequent increase in intracellular cAMP [14]. To confirm the lack of RAMP2 in the plasma membrane of TECs and thymocytes, observed with confocal microscopy, we evaluated cAMP levels in these cells following exposure to AM. In agreement with morphological results, treatment of human TECs and thymocytes with AM (10^−9^ to 10^−7^) for 10 minutes did not increase cAMP levels (Fig. 9), whereas the adenylate cyclase stimulator forskolin, used as a positive control in both cell lines, caused a 100- fold increase (p<0.05).

## Discussion

In our previous studies we observed that AM, RAMP2 and RAMP3 are expressed in rat thymus and investigated the role for AM and its AM1 and AM2 receptors in the control of thymic functions. In particular, we observed that AM exerts a potent growth promoting effect on rat thymocytes by enhancing the proliferation and lowering the apoptotic death of these cells [16], [17]. The present study demonstrates for the first time that AM and RAMP2, but not RAMP3, are largely distributed in the newborn human thymus, where they can be found in blood vessels, mast cells and the epithelial compartments of both the cortex and the medulla. Thus, our findings essentially confirm the results obtained in animal studies and expand them by an analysis of the localization of AM and the AM1 receptor proteins RAMP2 and CLR in human TECs and thymocytes.

It is worth noting that all cell cultures examined in this study were obtained from thymic fragments in which both cortex and medulla were represented; therefore our cultures always included both cTECs and mTECs. Almost all cultured TECs have been found to express AM, CLR and RAMP2. The distribution pattern of AM in TECs (cytoplasmic clusters) is similar to that previously observed in mammalian skin glands [24] and mast cells [18], [25], where AM is packaged in secretory granules and acts as an autocrine/paracrine signalling peptide. Thus, it can be speculated that AM may also have a similar signalling role in TECs, although functional studies are needed to confirm this hypothesis. As already demonstrated in other cell lines [26], [27], RAMP2 is present in the cytoplasm of TECs. However, contrary to what was observed with other cell lines [15], [26], [28], it also localizes to the nucleus, not to the cell membrane of TECs. The cytoplasmic and nuclear localizations of RAMP2 have been proved by both immunocytochemistry coupled to confocal fluorescence microscopy and western blot analysis of isolated cell fractions. The lack of RAMP2 in the plasma membrane, which prevents the formation of a functional AM1 receptor with CLR, has been confirmed by the absence of an increase in intracellular cAMP production in response to AM exposure. The finding that CLR and RAMP2 are both present in the cytoplasm and nucleus of TECs suggests that the AM1 receptor has an intracellular localization in these cells. The expression of CLR in the plasma membrane is not incompatible with the absence of RAMP2, since CLR also associates with RAMP1 to form the membrane-associated receptor for CGRP [13], which has actually been found in the plasma membrane of TECs [29]. The intracellular localization of a 7-transmembrane GPCR, such as AM1, is not surprising since an increasing number of GPCRs have been shown to be present in subcellular organelles in addition to their canonical localization in the plasma membrane. These organelles include the Golgi apparatus, the endoplasmic reticulum, the cytoskeleton, and the nuclear membrane (see [30]–[32] for reviews). Nuclear GPCRs have been demonstrated or suggested to regulate various physiological processes such as cell proliferation and survival, DNA synthesis and transcription, and inflammatory responses [31]. Since AM has been shown to regulate thymus growth and differentiation in the rat [16], [17], the possibility may be considered that AM may also exert a control of these functions in human thymus through the interaction with a receptor localized to the nucleus.

In contrast to TECs, which were all immunolabeled by AM, RAMP2 and CLR antibodies in each preparation, the expression of AM and its AM1 receptor could be observed only in some thymocytes, in which they displayed a variable intracellular localization with a different intensity of expression. Different subsets of thymocytes are present in thymus, where they constitute intermediary stages of the T-cell differentiation pathway [3]. The maturation stage of these cells can be detected by following the CD4 and CD8 surface expression: thymocyte precursors (CD4^−^ CD8^−^, called “double negative” thymocytes) become cortical thymocytes (CD4^+^ CD8^+^, “double positive”) and then mature medullary cells (“single positive”, CD4^+^ CD8^−^ or CD4^−^CD8^+^) [33]. Since not all thymocytes are immunopositive for CLR, RAMP2 and AM, the possibility can be considered that the expression of AM, CLR and RAMP2 is dependent on the maturation stage of thymocytes. T cell development and selection are not cell-autonomous processes, but developing T cell precursors require a constant input from TECs [3] and, conversely, the functional maturation of TECs is strictly dependent on instructive signals provided by thymocytes, which constitutes the symbiotic bidirectional “thymic cross-talk” [1], [10]. It is well known that this form of communication is regulated by cytokines and/or hormones that are locally secreted [3]. Since AM is known to regulate cytokine secretion, for example in skin epithelial cells [34], microglia [35] and macrophages [36], it cannot be excluded that AM may play a role in thymic cross-talk. The role of AM in regulating cytokine secretion by TECs and thymocytes is currently investigated in our laboratory.

The presence of AM and RAMP2 in newborn thymic blood vessels is not unexpected, since the AM system is largely represented in all kinds of blood vessels, where it participates in the regulation of vascular tone, permeability and regeneration, acting as a proangiogenic factor in both physiological and pathological conditions [37], [38]. The perivascular localization of AM-storing mast cells is consistent with the important paracrine role exerted by these cells in the regulation of blood circulation, in which they can release vasoactive peptides, such as AM [18], [25], [39].

In conclusion, the present data provide the first evidence that: 1) AM and the AM1 proteins CLR and RAMP2 are expressed in human thymus from newborns; 2) contrary to what has been observed in other cell lines, the AM1 receptor is not located in the plasma membrane, but its two components CLR and RAMP2 are present in the cytoplasm and nucleus of both TECs and thymocytes. This is consistent with recent observations [31], [32] that a peptidergic GPCR can localize to the nuclear membrane and suggests that AM may participate in the regulation of thymic functions, such as thymocyte apoptosis and proliferation, by interacting with an intracellular receptor. Functional studies are in progress in our laboratory in order to clarify the AM role in human thymus.

## Supporting Information

Figure S1 **TEC culture purity.** Immunostaining for the epithelial cell marker Ck 8/18, assessing TEC culture purity. When, for control purposes, the primary antibody was replaced by a non-immune PBS solution, no reactivity could be observed (not shown in the figure). Original magnification ×10 A; ×60 B.
(TIF)

Figure S2 **RAMP2 distribution in cultured TECs.** Immunofluorescence staining of TECs for RAMP2 (green) with an alternative antibody directed against the extracellular N-terminus, the region which determines the binding phenotype. Cell nucleus is red stained with propidium iodide. When, for control purposes, the primary antibody was replaced by a non-immune PBS solution, no reactivity could be observed (not shown in the figure).
(TIF)
