# Supplementary figures and images for: Expression and Distribution of the Adrenomedullin System in Newborn Human Thymus
Source: PLoS One. 2014 May 15;9(5):e97592. doi: 10.1371/journal.pone.0097592 (PMC4022580; doi:10.1371/journal.pone.0097592)

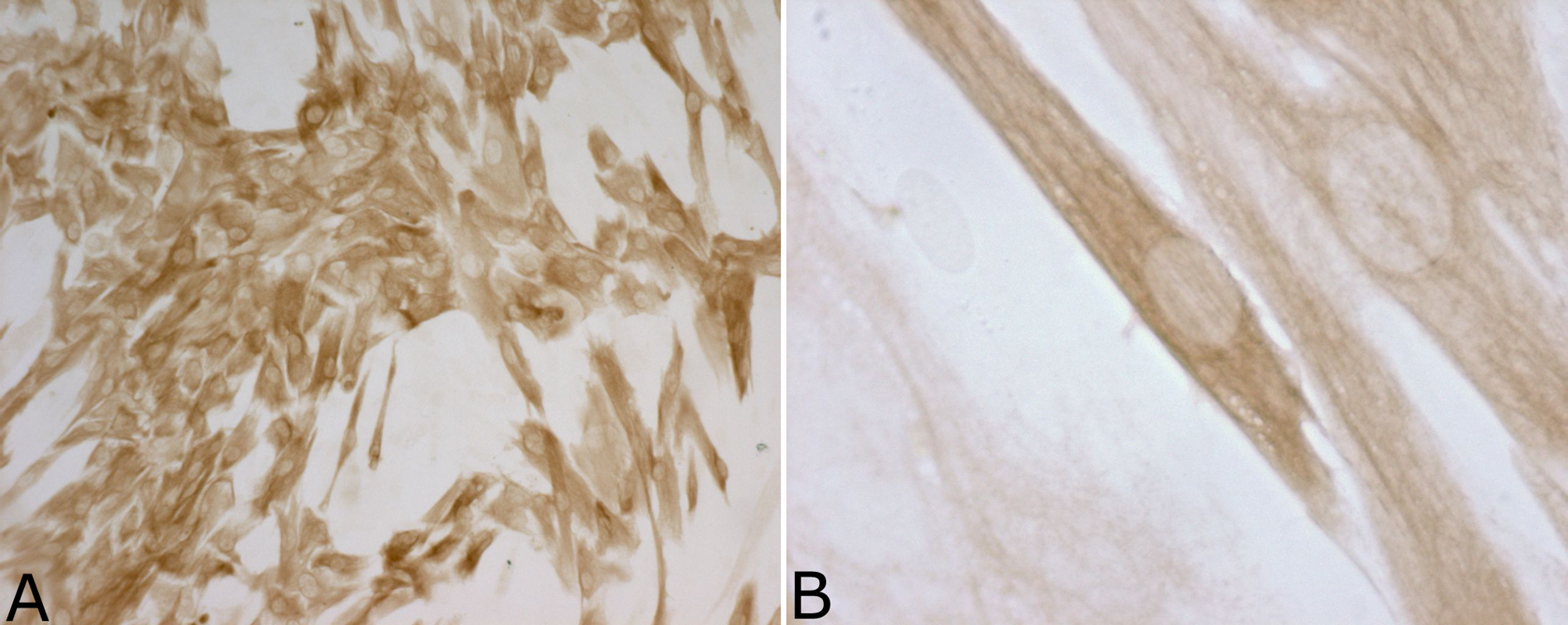

Supplement: Figure S1 — TEC culture purity. Immunostaining for the epithelial cell marker Ck 8/18, assessing TEC culture purity. When, for control purposes, the primary antibody was replaced by a non-immune PBS solution, no reactivity could be observed (not shown in the figure). Original magnification ×10 A; ×60 B. (TIF) [file pone.0097592.s001.tif]

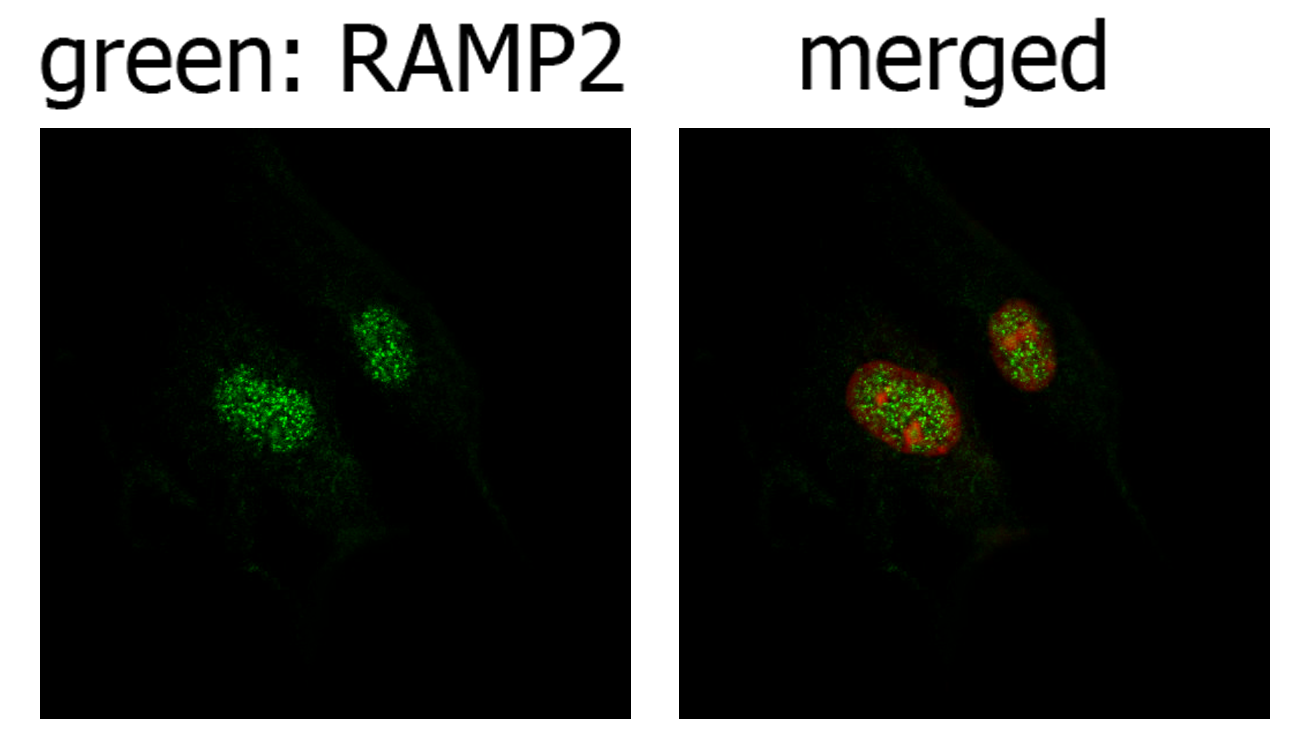

Supplement: Figure S2 — RAMP2 distribution in cultured TECs. Immunofluorescence staining of TECs for RAMP2 (green) with an alternative antibody directed against the extracellular N-terminus, the region which determines the binding phenotype. Cell nucleus is red stained with propidium iodide. When, for control purposes, the primary antibody was replaced by a non-immune PBS solution, no reactivity could be observed (not shown in the figure). (TIF) [file pone.0097592.s002.tif]
